# Genome-Wide Identification and Analysis of the Plant Cysteine Oxidase (PCO) Gene Family in *Brassica napus* and Its Role in Abiotic Stress Response

**DOI:** 10.3390/ijms241411242

**Published:** 2023-07-08

**Authors:** Xiaohua Bian, Yifan Cao, Ximin Zhi, Ni Ma

**Affiliations:** 1 Oil Crops Research Institute, Chinese Academy of Agricultural Sciences, Wuhan 430062, China; biobxhbxh@163.com (X.B.); 15137633430@163.com (Y.C.); 82101219205@caas.cn (X.Z.); 2 College of Plant Science and Technology, Huazhong Agricultural University, Wuhan 430070, China

**Keywords:** *Brassica napus*, Plant Cysteine Oxidase (PCO), gene expression, abiotic stress

## Abstract

Plant Cysteine Oxidase (PCO) is a plant O_2_-sensing enzyme catalyzing the oxidation of cysteine to Cys-sulfinic acid at the N-termini of target proteins. To better understand the *Brassica napus PCO* gene family, *PCO* genes in *B. napus* and related species were analyzed. In this study, 20, 7 and 8 *PCO* genes were identified in *Brassica napus*, *Brassica rapa* and *Brassica oleracea*, respectively. According to phylogenetic analysis, the *PCOs* were divided into five groups: *PCO1*, *PCO2*, *PCO3*, *PCO4* and *PCO5*. Gene organization and motif distribution analysis suggested that the *PCO* gene family was relatively conserved during evolution. According to the public expression data, *PCO* genes were expressed in different tissues at different developmental stages. Moreover, qRT-PCR data showed that most of the *Bna/Bra/BoPCO5* members were expressed in leaves, roots, flowers and siliques, suggesting an important role in both vegetative and reproductive development. Expression of *BnaPCO* was induced by various abiotic stress, especially waterlogging stress, which was consistent with the result of cis-element analysis. In this study, the *PCO* gene family of *Brassicaceae* was analyzed for the first time, which contributes to a comprehensive understanding of the origin and evolution of *PCO* genes in *Brassicaceae* and the function of *BnaPCO* in abiotic stress responses.

## 1. Introduction

Cysteine (Cys) oxidation is an essential post-translational modification (PTM), which controls protein half-life and protein function [1]. It is reported that ROS-mediated hormone signaling can be transduced by cysteine oxidation in plant, which is associated with root growth, pollen tube elongation and various abiotic stress responses [2]. Plant Cysteine Oxidases (PCOs) are the enzymes catalyzing N-terminal cysteinyl residues to sulfinic acid to promote proteasomal degradation in plants [3]. It has been shown that Plant Cysteine Oxidases use molecular oxygen atoms to catalyze dioxygenation of Nt-Cys to Cys-sulfinic acid in ERF-VIIs (ETHYLENE RESPONSE FACTOR group VIIs), as well as other substrates VRN2 and ZPR2 [4,5] for subsequent Nt-arginylation [6,7]. Meanwhile, ERF-VIIs are known as the regulators of hypoxia-regulated transcriptional reprogramming to adapt the environmental change [8,9,10]. PCO activity is sensitive to physiologically relevant fluctuations in O_2_ availability [11], thus these enzymes can act as plant O_2_ sensors with a key role in regulating ERF-VII stability. In other words, as an enzyme, Plant Cysteine Oxidases (PCOs) are a direct link between environmental stimuli and molecular physiological outcomes [12].

In Arabidopsis, AtPCOs catalyze the oxidation of cysteine to Cys-sulfinic acid at the N-termini of target proteins, a reaction that co-translational methionine cleavage exposes the N-terminal Cys for oxidation [6,7,11]. Oxidized N-terminal Cys residues are substrates for arginyl transferase enzymes, with the arising arginylated N-termini recognized by ubiquitin ligases [13]. Additionally, then the ubiquitinated protein will be degraded. Therefore, the N-termini is the signal for protein degradation, which is called the N-degron pathway. It is reported that PCOs isolated from *Marchantia polymorpha* (MpPCO) and *Klebsormidium nitens* (KnPCO) exhibit cysteine dioxygenase activity, indicating that PCO enzymes are conserved in early land plants and algae [14]. The MpPCO was incubated with the N-termini of MpERF-like (a 14-mer peptide representing the Cys-initiating N-terminus of MpERF-like, CRMNKRLGKGETGL), and MpPCO-catalyzed MpERF-like oxidation reached 89.3% after 1 h. Meanwhile, homologs of arginyl-tRNA transferase (ATE) and E3 N-recognin, PROTEOLYSIS (PRT) 6 can be found in *Marchantia polymorpha*, suggesting the conserved way of PCO in catalyzing the oxidation of cysteine to Cys-sulfinic acid at the N-termini of target proteins.

As PCOs regulate ERF-VIIs levels by catalyzing cysteine oxidation in N-degron pathway and ERF-VIIs activate the anaerobic gene expression of *Alcohol Dehydrogenase* (*ADH*), *Pyruvate Decarboxylase* (*PDC1*) and *Hypoxia Responsive Attenuator 1* (*HRA1*), disrupting the N-degron pathway of ERF-VIIs in barley shows altered seed germination and enhanced yield under waterlogging stress. Thus, it is critical to uncover PCO structures to manipulate their enzyme activities for crop improvement [15]. The structures of AtPCO4_1, AtPCO4_2 (two different structures of AtPCO4 from independent crystallization conditions) and AtPCO5 are resolved to 1.82, 1.24 and 1.91 Å resolutions [12]. There is a core double-stranded beta-helix (DSBH) supporting three histidine residues to coordinate the active site metal ions involved in catalysis. To recognize the active site, the Tyr_182_-Ser_183_-Ser_184_-Glu_185_-His_186_-Asp_187_-Arg_188_-His_189_-Cys_190_ fragment is characterized by targeted mutagenesis [12]. AtPCO4 variant C190A shows the same enzymatic activities as the wild type, but the enzyme activities of variants Y182F, H164D and D176N are reduced to 60%, 0, and 5%, respectively. Furthermore, expression of AtPCO4 H164D and D176N lead to strongly increase the anaerobic gene expression, which indicates the catalytic function of enzyme is invalid when the enzyme activity is lower than 5%. Therefore, the sites (amino acid 160–190 position) of PCO4 play an important role in the enzyme activity [12,16,17], and modification of enzyme activity by site-directed mutagenesis of the enzyme active sites can be used for crop improvement.

Studies show that single, double or triple (*pco1/2/4*) mutants show similar phenotype with wild type and double mutant *pco4/5* increases the resistance to anoxic stress in Arabidopsis [18]. However, quadruple *pco1/2/4/5* mutant plants (*4pco*) show pleated, pale leaves with extensive serration and male and female sterility, along with constitutive expression of low-oxygen response genes [5,7,12]. Mutations of AtPCO4 (H164D) and AtPCO4 (D176N), which destroy the chemically reactive residues, fail to reconstitute the wild-type phenotype in *4pco* mutant. On the other hand, overexpression of *AtPCO1* or *AtPCO2* in Arabidopsis show smaller leaves and decreased biomass [7]. It suggests that an appropriate PCO level or enzyme activity is the fundamental to maintain plant growth and development. The PCOs catalyze the reaction of oxygen with the conserved N-terminal cysteine of ERF-VIIs to form cysteine sulfinic acid, triggering degradation under normal conditions. PCO activity decreases under hypoxia conditions and then the stabilized ERF-VIIs activate the expression of anaerobic genes (*ADH*, *PDC1*, *HRA1*) in response to stress [6,19]. Previous studies have shown that aerobic or hypoxia related genes are down-regulated in either *AtPCO1* or *AtPCO2* over-expressed plants, whereas anaerobic genes are constitutively up-regulated in quadruple *pco1/2/4/5* mutants [13]. Compared with wide type, plants over-expressing *AtPCO1* or *AtPCO2* show sensitive to prolonged submergence stress [7]. Meanwhile, overexpression of the ERF-VII *RAP2.12* in Arabidopsis results in improved tolerance to submergence and up-regulation of genes associated with the hypoxic response [10,15]. Therefore, PCOs, as an O_2_ sensor, play a critical role in stress response, especially in hypoxia stress.

Approximately 7500 years ago, a natural heterotetraploid *Brassica napus* (AACC, 2n = 38) was formed by the hybridization of *Brassica rapa* (2n = 20, AA) and *Brassica oleracea* (2n = 18, CC) [20,21,22]. Rapeseed (*Brassica napus*) is an important oil crop, with the largest planting area as well as total oil production in China. Rapeseed is mainly distributed in the Yangtze River basin in China and waterlogging, caused by the rainy and humid climate in this region, is a common abiotic stress affecting rapeseed production. The oxygen availability in waterlogging soil is greatly limited and the yield of rapeseed could decrease by 17–42% due to waterlogging stress in China [23]. Research on genetics, evolution and stress response of rapeseed is a good way to improve the quality and yield of rapeseed [24,25,26,27,28]. While *AtPCOs* are expected to play important roles in stress responses, detailed genome-wide analysis of the *PCO* gene family in *Brassicaceae* has not been performed. In this study, we investigated the significant role of *PCO* genes in *Brassicaceae* to provide evidence for resistance improvement of rapeseed. As a result, we identified 35 *PCO* genes of *Brassica napus*, *Brassica rapa* and *Brassica oleracea*, respectively, and characterized the gene structures, chromosomal locations, evolutionary relationships and expression patterns in different tissues and under different abiotic stress treatments with public data analysis. Moreover, we confirmed the *BnaPCO* gene expression patterns in different tissues and under waterlogging stress by qRT-PCR analysis. Therefore, this study provides a comprehensive understanding of *PCOs* in development and abiotic stress responses in *Brassica napus*.

## 2. Results

### 2.1. Identification and Classification of PCO Genes in B. napus, B. oleracea and B. rapa

To identify PCO proteins in *B. napus*, *B. oleracea* and *B. rapa*, we performed a BLASTp search against the annotated proteins of *B. napus* (Zhongshuang 11 variety), *B. oleracea* and *B. rapa* in BRAD (http://www.brassicadb.cn/#/BLAST/, accessed on 10 January 2023) and BnIR (https://yanglab.hzau.edu.cn/BnIR, accessed on 15 June 2022) using Arabidopsis AtPCO protein (AtPCO1, AtPCO2, AtPCO3, AtPCO4 and AtPCO5) sequences as queries. Sets of 20 (4 BnaPCO1, 2 BnaPCO2, 6 BnaPCO3, 4 BnaPCO4 and 4 BnaPCO5), 8 (2 BoPCO1, 1 BoPCO2, 1 BoPCO3, 2 BoPCO4 and 2 BoPCO5) and 7 (2 BraPCO1, 1 BraPCO2, 1 BraPCO3, 1 BraPCO4 and 2 BraPCO5) PCO proteins were identified in *B. napus*, *B. oleracea* and *B. rapa*, respectively (Table 1). All 35 *PCOs* in *B. napus*, *B. oleracea* and *B. rapa* encoded the amino acids ranging in length from 82 to 308 with the molecular weight from 8650 to 34,180 Dalton. The isoelectric point (pI) of these amino acids ranged from 4.39 to 8.78 (Table 1).

**Figure 1:**
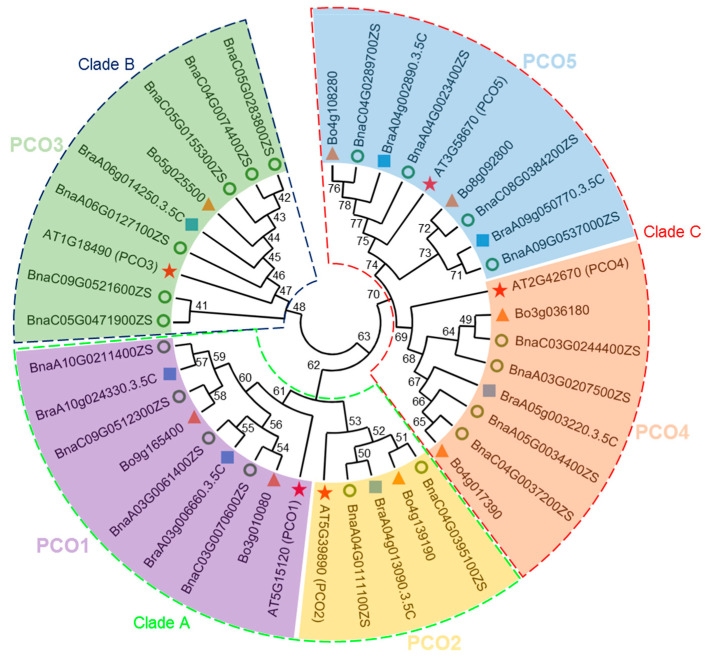
Phylogenetic analysis of 40 PCO proteins from *B. napus* (20), *B. rapa* (7), *B. oleracea* (8) and Arabidopsis (5). The green circle represents the protein from *B. napus*. The blue square represents the protein from *B. rapa*. The yellow triangle represents the protein from *B. oleracea*. The red star represents the protein from Arabidopsis.

To explore the classification and evolutionary characteristics of the PCO proteins, an unrooted phylogenetic tree based on the 40 PCO protein sequences of *B. napus* (20), *B. oleracea* (8) and *B. rapa* (7) and Arabidopsis (5) was constructed in MEGA 11 (Figure 1). According to the phylogenetic analysis of PCO proteins, there are five groups: *PCO1* (homologous to *AT5G15120.1*/*AtPCO1*), *PCO2* (homologous to *AT5G39890.1*/*AtPCO2*), *PCO3* (homologous to *AT1G18490.1*/*AtPCO3*), *PCO4* (homologous to *AT2G42670.1*/*AtPCO4*) and *PCO5* (homologous to *AT3G58670.1*/*AtPCO5*). In addition, according to the phylogenetic tree, PCO proteins can be clearly divided into three clades: PCO1s and PCO2s belong to clade A, PCO3s belong to clade B, and PCO4 and PCO5 belong to clade C, respectively. 

### 2.2. Chromosomal Distribution and Duplication of BnaPCOs

**Figure 2:**
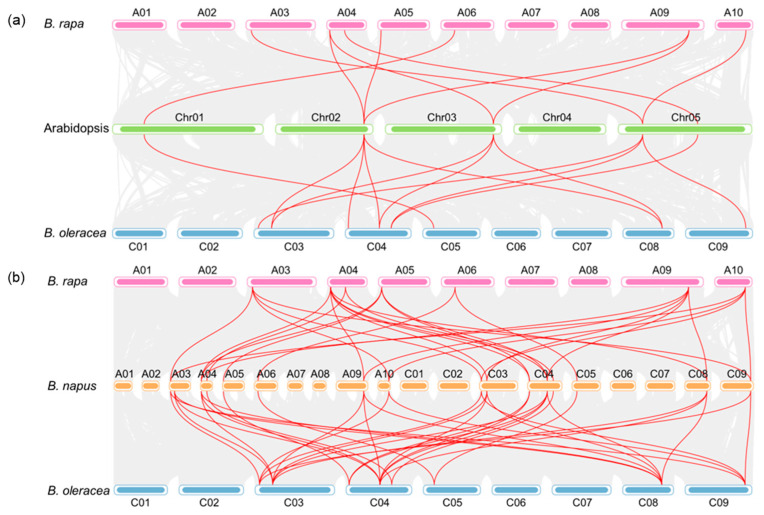
Syntenic relationship of *PCO* genes in *B.napus* and three ancestral plant species. (**a**) Syntenic relationship of *PCO* genes in Arabidopsis, *B. rapa* and *B. oleracea*. (**b**) Syntenic relationship of *PCO* genes in *B. napus*, *B. rapa* and *B. oleracea*. Grey lines in the background show the collinear blocks within rapeseed and other plant genomes, while the red lines highlight the syntenic *PCO* gene pairs.

It was uncovered that *B. napus* (AnAnCnCn, 2n = 38) originated from the hybridization event between *B. rapa* (AnAn, n = 10) and *B. oleracea* (CnCn, n = 9) approximately 7500 years ago [20,21,22], and *Brassicaceae* species underwent a whole-genome triplication event [20]. As shown in Figure 2a, three genes in *B. rapa* were oriented from *At2G42670*, which was consistent with the duplication theory. According to the evolution theory, there are 15, 15 and 30 *PCOs* in *B. rapa*, *B. oleracea* and *B. napus* expanded from the 5 *AtPCOs*. However, only 7, 8 and 20 genes were identified in *B. rapa*, *B. oleracea* and *B.napus*, respectively (Table S1). As shown in Figure 2, 7 *BraPCOs* were scattered on 6 of the 10 *B. rapa* chromosomes, 8 *BoPCOs* were scattered on 5 of the 9 *B. oleracea* chromosomes, 20 *BnaPCOs* were scattered on 11 of the 19 *B. napus* chromosomes. The numbers of *BnaPCOs* on the An-subgenome (8 genes) and the Cn-subgenome (12 genes) showed a biased trend, with more genes on the Cn-subgenome. It was indicated that some *PCOs* may have been lost during evolution. 

Previous study reported that the An and Cn subgenomes of *B. napus* were collinear with the diploid *B. rapa* (Ar) and *B. oleracea* (Co) genomes, and most of the An-Ar and Cn-Co homologous pairs showed similar chromosomal locations [20]. However, the C genome of *B. napus* had more homologous genes corresponding to the *BoPCO3* gene (*Bo5G025500*), and the A genome had more homologous genes corresponding to the *BraPCO4* gene (*BraA05G003220*), while the homologous genes of *PCO1*, *PCO2* and *PCO5* groups were relatively conserved (Table 1). It was inferred that the *PCO* sequences were mutated or duplicated in the homologous evolution process.

### 2.3. Gene Structures and Motif Analysis of PCOs in B. napus

**Figure 3:**
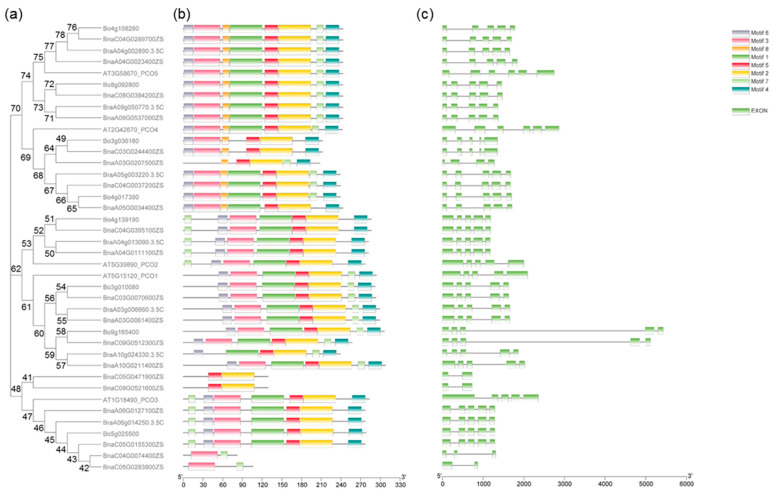
Phylogenetic tree (**a**), gene motif (**b**) and gene structure (**c**) of *PCO* of Arabidopsis, *B. napus*, *B. rapa* and *B. oleracea*. (**a**) Neighbor-joining phylogenetic tree showing the relationship among 5 Arabidopsis, 20 *B. napus*, 7 *B. rapa* and 8 *B. oleracea* PCO proteins. (**b**) Eight motifs in PCO proteins were identified by MEME tools. (**c**) Green box indicates the exon regions on *PCO* genes.

In order to study the homology domain and conservation degree of the *BnaPCOs*, MEME [29] and TBtools [30] were used to predict and visualize their conserved domain and gene organization, respectively. 8 motifs were predicted by MEME (https://meme-suite.org/meme/tools/meme, accessed on 10 April 2023) (Figure S1). As shown in Figure 3, Motif 2 and 5 were the most conserved, with 38 of the 40 genes containing these two motifs. All of the PCO4 and PCO5 proteins contained Motif 8, whereas none of the other genes contained Motif 8. All PCO5 proteins contained eight motifs, of which the Motif 7 was located at the C terminal. However, all PCO2 proteins contained seven motifs in addition to the Motif 8, with the Motif 7 located at the N terminal. Two of the four short abnormal proteins, BnaC05G0471900ZS and BnaC09G0521600ZS, lacked Motif 1-3-4-6-7-8, while BnaC04G0074400ZS and BnaC05G0283800ZS lacked Motif 1-2-4-5-6-8. Further protein analysis exhibited that BnaC05G0471900ZS and BnaC09G0521600ZS aligned C-terminal of BnaPCO3 protein and BnaC04G0074400ZS and BnaC05G0283800ZS aligned N-terminal of BnaPCO3 protein (Figure S8).

It is reported that some introns play an essential role in transcriptional regulation [31]. We also investigated the distribution of introns and exons to study the diversity of gene structure. Five exons and four introns were distributed on most of *PCO* genes. Additionally, the intron phases of *BnaPCOs* were highly conserved in the same group, implicating the evolutionary similarity between these members.

### 2.4. Cis-Element Analysis of BnaPCOs

Cis-elements regulate the initiation and efficiency of gene transcription by binding to transcription factors [32]. We analyzed the cis-elements of 20 *PCOs* promoters with PlantCARE [33]. Cis-elements of plant growth and development, hormone response and abiotic stress response were identified in *BnaPCOs* promoter region (Table S2). The identified environmental stress-related elements included anaerobic induction, circadian control, defense and stress responsive, drought induction, light response, low temperature response, meristem expression, and wound response. Among them, the most common elements were associated with light response and anaerobic induction, indicating that the growth and development of plants regulated by *BnaPCOs* was affected by light and oxygen. *PCOs* was known as a sensor of oxygen, which was consistent with the cis-elements result. However, the light response of *PCOs* needed further study.

**Figure 4:**
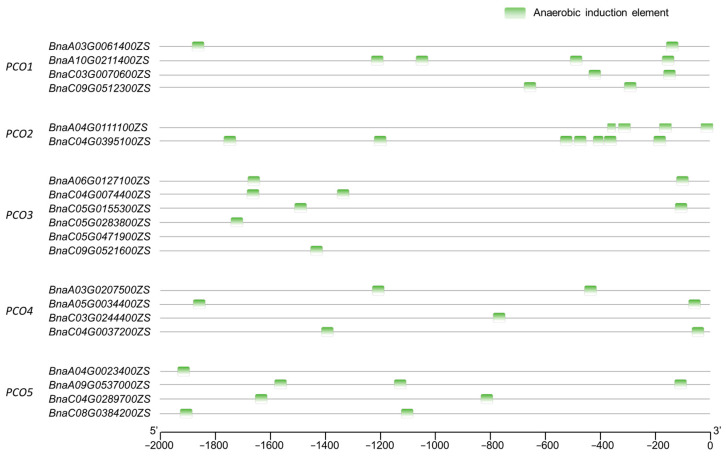
Anaerobic-induction cis-elements distributed on the *BnaPCOs* promoter regions. Cis-elements were identified by PlantCARE.

In addition, there were more anaerobic-induction elements on *PCO2s* promoter region compared with other genes and there was no anaerobic-induction elements on the promoter of *BnaC05G0471900ZS* (belongs to *BnaPCO3*) (Figure 4). The cis-elements in the upstream promoter region of genes are closely related to the expression and function of downstream genes [34]. Therefore, according to the anaerobic-induction elements result, it indicated that the expression of *PCO2s* may be highly induced by hypoxia and it also implies the diversification of biological functions of *PCO* genes in *B. napus*.

### 2.5. Expression Profiling of PCO Genes in Different Tissues

**Figure 5:**
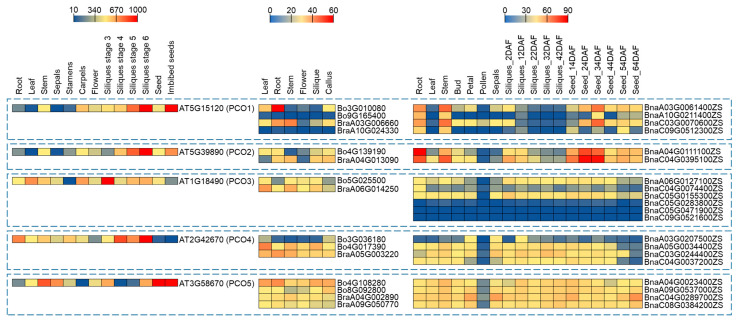
Expression of *AtPCO*, *BraPCO*, *BoPCO* and *BnaPCO* (TPM values, Transcripts Per Kilobase Million, public expression data [35,36,37]) in different tissues. The expression levels of *PCO* genes are indicated by differently colored rectangles. DAF represents day after flower.

**Figure 6:**
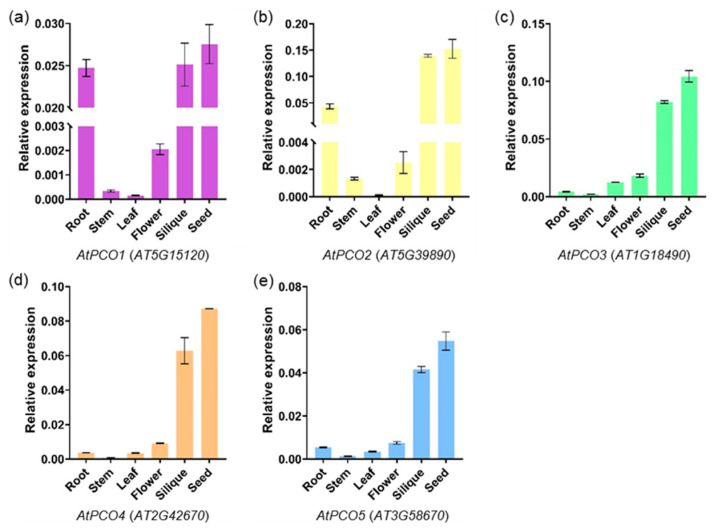
Expression pattern of *AtPCOs* in different tissues by qRT-PCR assays. *AtPCO1* ((**a**), purple), *AtPCO2* ((**b**), yellow), *AtPCO3* ((**c**), green), *AtPCO4* ((**d**), orange) and *AtPCO5* ((**e**), blue) are represented in different colors. The mRNA levels were normalized to *AtActin2*. Bars indicate ± SD (n = 3).

**Figure 7:**
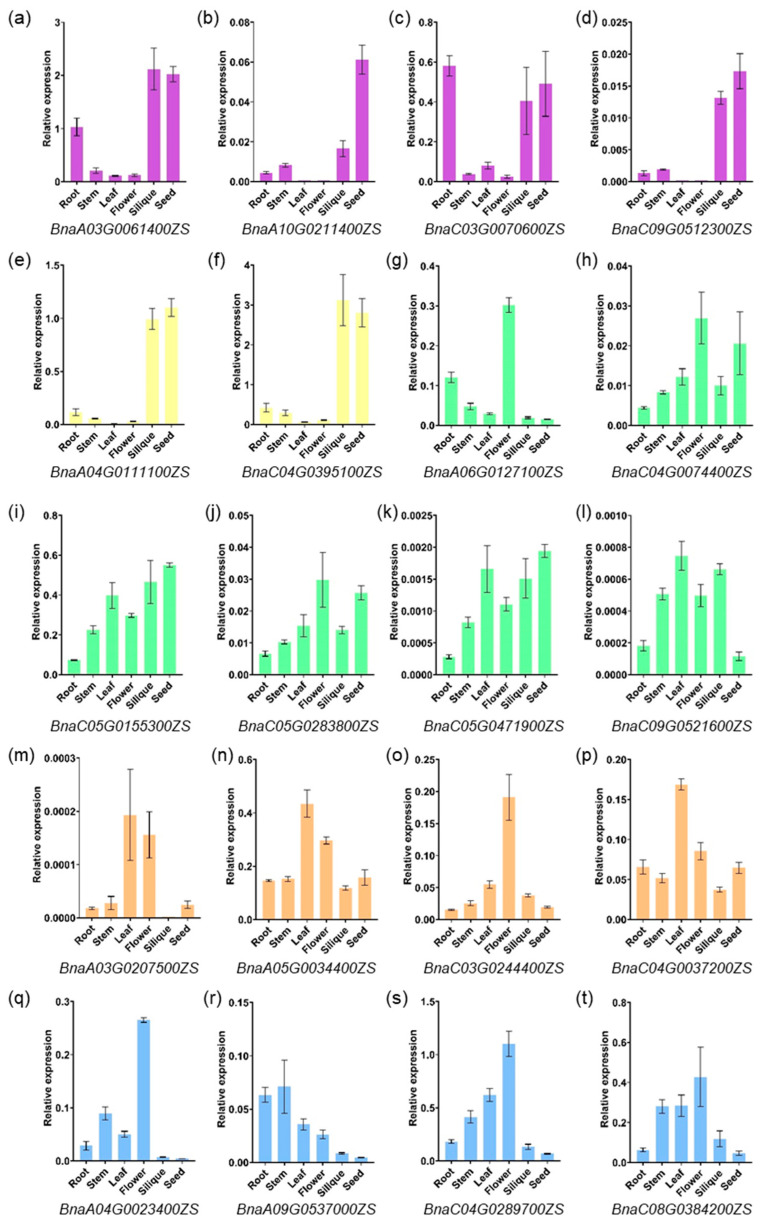
Expression pattern of *BnaPCOs* in different tissues by qRT-PCR assays. *BnaPCO1* ((**a**–**d**), purple), *BnaPCO2* ((**e**,**f**), yellow), *BnaPCO3* ((**g**–**l**), green), *BnaPCO4* ((**m**–**p**), orange) and *BnaPCO5* ((**q**–**t**), blue) are represented in different colors. The mRNA levels were normalized to *BnaActin7*. Bars indicate ± SD (n = 3).

Based on Arabidopsis eFP Browser data (http://bar.utoronto.ca/efp/cgi-bin/efpWeb.cgi, accessed on 28 March 2023) and RNA-seq data (*B. rapa*: GSE43245, *B. oleracea*: GSE42891 and *B. napus*: BnIR database) (Table S3), the *PCO* genes were expressed in different vegetative and reproductive organs of the four species at different developmental stages (Figure 5). qRT-PCR was performed to verify the expression pattern in Arabidopsis, *B. rapa*, *B. oleracea* and *B. napus* (Figure 6, Figure 7 and Figure S9). In general, the expression pattern of *PCO* differed between groups (Figure 5). Almost all *PCOs* were weakly expressed in pollen, indicating that *PCO* expression was down-regulated in sperm cells, presumably due to chromosomal structure or histone modifications. On the other hand, most of *PCO5s* were expressed in different tissues, indicating that it involved in both vegetative and reproductive development. Compared with the public data, qRT-PCR analysis of *BnaPCOs* showed the similar results (Figure 7).

There were six *PCO3* genes in *B. napus*, and genes (*BnaA06G0127100* and *BnaC05G0155300*) with higher sequence similarity to ancestral genes (*BraA6G014250* and *Bo5G025500*) had higher expression levels, whereas genes with lower sequence similarity to ancestral genes had lower expression levels (Figure 7 and Figure S4). This suggested that genes with higher sequence similarity to the ancestral genes may play a major role, while the new genes may be pseudogenes that have been amplified during evolution or may be silent under normal conditions and be as a backup for their homolog genes under special conditions.

### 2.6. Expression Profiling of PCO Genes under Abiotic Stress Treatment

**Figure 8:**
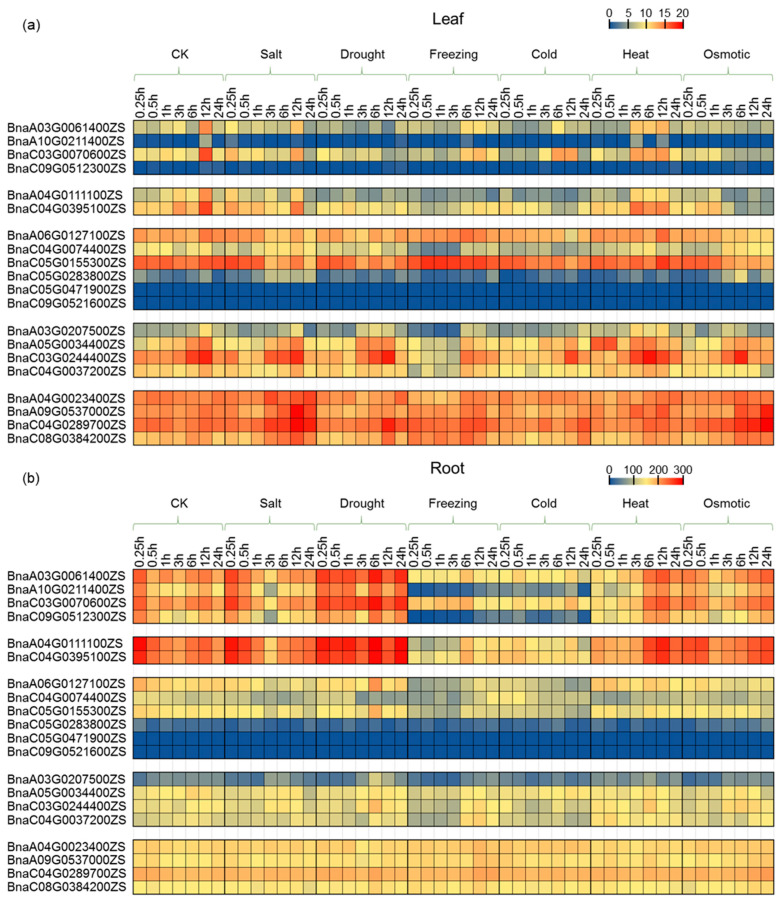
Expression of *BnaPCO* genes under different abiotic stress treatments. The expression levels (TPM values, Transcripts Per Kilobase Million, public expression data [37]) of *BnaPCO* genes are indicated by differently colored rectangles. (**a**) Gene expression represents the expression in leaves. (**b**) Gene expression represents the expression in roots.

To reveal the roles of *PCOs* in stress response in *B. napus*, the expression patterns upon various abiotic treatment were investigated (Figure 8, Table S4). In general, the expression of most *PCO* genes in leaves did not change significantly under various abiotic stress treatments (Figure 8a). In leaves, *PCO3* gene expression was strongly induced under freezing stress, and *PCO5* gene expression was increased under salt and osmotic stress as well. However, the response of *PCO* genes to stress was much stronger in roots than in leaves (Figure 8b). In roots, the expression of *PCO1* and *PCO2* were strongly induced by drought, and the expression of *PCO1*, *PCO2*, *PCO3* and *PCO4* were extremely down-regulated by freezing and cold stress. However, *PCO5* gene expression in roots changed weakly under the abiotic stress treatments compared with other *PCO* genes, which was consistent with the hypothesis that *PCO5* worked as a fundamental gene.

Though there were similar motifs of drought-inducible and low-temperature responsive on *PCOs* promoter region, the gene expression pattern was various under drought, freezing or cold stress treatment. It implied that there were other regulators along with cis-elements to regulate *PCO* gene expression. It was interesting that *PCO3* was up-regulated in leaves and down-regulated in roots after freezing treatment. In other words, *PCO3* showed opposite response patterns in leaves and roots under freezing stress treatment. This suggested that *PCO3* may play different roles in leaves and roots under freezing stress, and further studies were needed to clarify this.

### 2.7. Expression Profiling of PCO Genes under Waterlogging Stress

**Figure 9:**
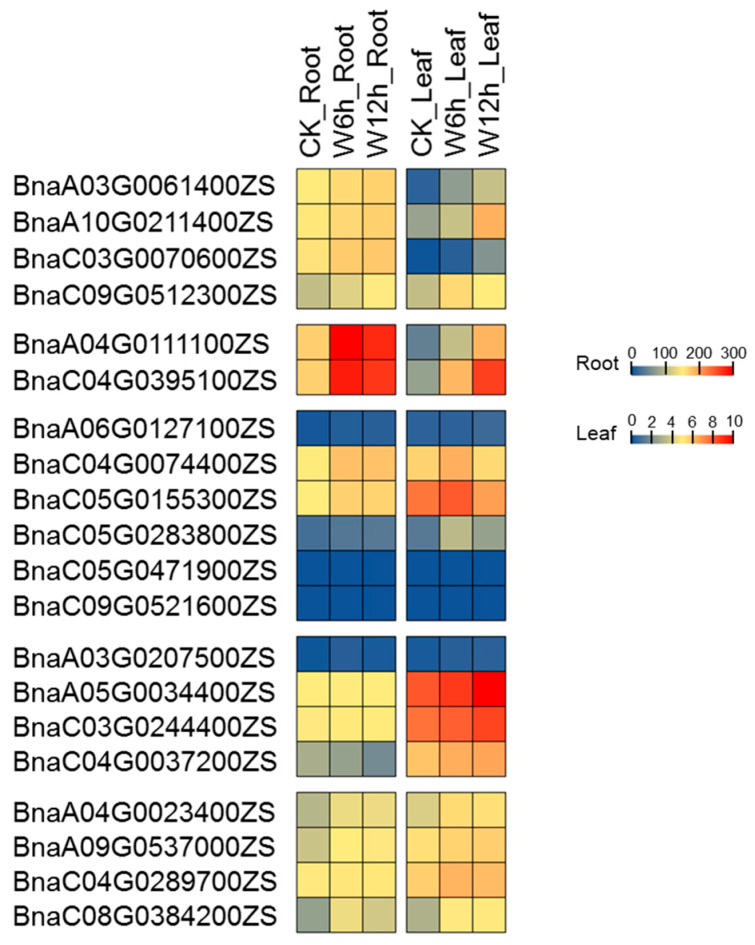
Expression of *BnaPCO* genes (FPKM, RNA-seq data) under waterlogging stress treatment for 6 h and 12 h. The expression levels of *BnaPCO* genes are indicated by differently colored rectangles. CK represents normal conditions. W represents waterlogging stress treatment.

**Figure 10:**
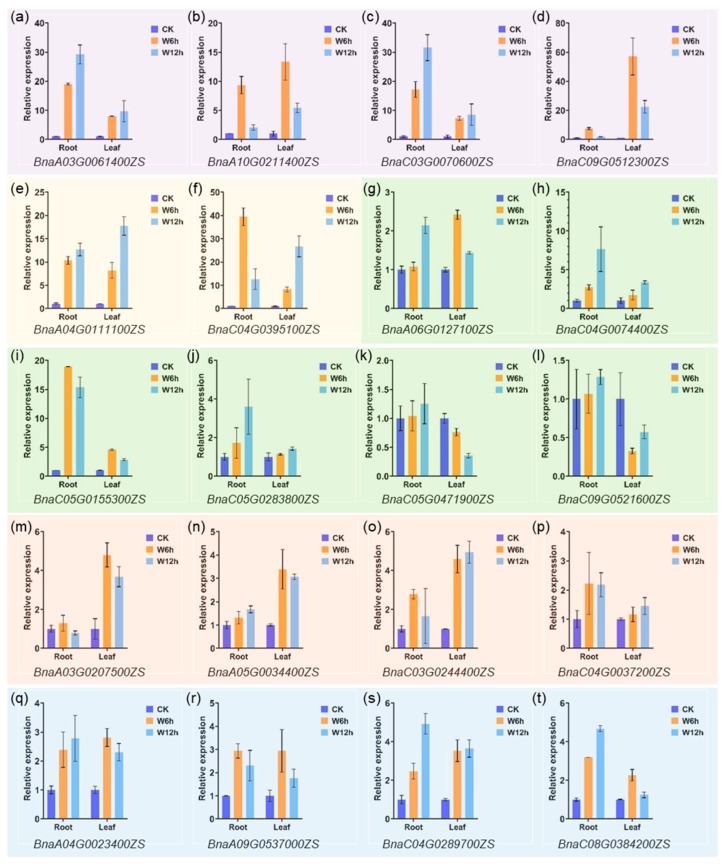
*BnaPCO* gene expression after waterlogging stress treatment for 6 h and 12 h by qRT-PCR assays. Genes with purple, yellow, green, orange and blue background represent *BnaPCO1* ((**a**–**d**)), *BnaPCO2* (**e**,**f**), *BnaPCO3* ((**g**–**l**)), *BnaPCO4* ((**m**–**p**)) and *BnaPCO5* ((**q**–**t**)) genes, respectively. CK represents normal conditions. W represents waterlogging stress treatment. The mRNA level (relative to *BnaActin7*) of each gene in CK-Root or CK-Leaf was set to 1. Bars indicate ± SD (n = 3).

Waterlogging removes air from soil leading to a blockage of gas exchange between the soil and the atmosphere [38,39]. Meanwhile, the diffusion rate of oxygen in water is only one tenth of that in air. As a result, oxygen availability in flooded soils is greatly limited, leading to suppression of root respiration. As mentioned before, *PCO* is an oxygen sensor in plant [11]. Therefore, it is speculated that *PCO* plays an important role in the hypoxic response induced by waterlogging stress. In order to elucidate the potential function of *BnaPCO* in response to waterlogging stress, RNA-seq and qRT-PCR assay were performed with leaves and roots after waterlogging stress in *B.napus*. Transcriptome data and qRT-PCR data (Figure 9 and Figure 10, Table S5) showed that compared with CK, the expression of *PCOs* was significantly induced by waterlogging stress. Compared to other genes, the expression of *PCO2* gene pairs (*BnaA04G0111100ZS* and *BnaC04G0395100ZS*) were strongly induced both in leaves and roots, while the expression of *PCO4* gene pairs (*BnaA05G0034400ZS* and *BnaC03G0244400ZS*) were particularly strongly expressed in leaves. In addition, gene expression of *PCO3* (*BnaC05G0471900ZS* and *BnaC09G0521600ZS*) were barely detectable in the transcriptome data, which was consistent with the previous result (Figure 5 and Figure 7). According to the expression results, the function of *PCO* was conserved under hypoxic stress.

## 3. Discussion

The Plant Cysteine Oxidase family (PCO) is a set of plant O_2_-sensing enzymes, which catalyze the O_2_-dependent step [11]. In Arabidopsis, *PCO* has five members, *PCO1*, *PCO2*, *PCO3*, *PCO4* and *PCO5*. In this study, 20, 8 and 7 *PCO* genes in *B. napus*, *B. oleracea* and *B. rapa* were identified, respectively (Figure 1). In *B. napus*, the number of *PCO* genes in the An subgenome (8) was almost the same as that in the diploid ancestors *B. rapa* (7) (Table 1). This showed that the An subgenome *PCO* genes were relatively preserved after the whole-genome duplication event in *B. napus*. However, *PCO3* genes in the Cn subgenome (*BnaC04G0074400ZS*, *BnaC05G0155300ZS*, *BnaC05G0283800ZS*, *BnaC05G0471900ZS* and *BnaC09G0521600ZS*) were mutated from the diploid ancestor *B. oleracea* (*Bo5G025500*). It suggested that the Cn subgenome was much flexible during the evolution, compared with the An subgenome [40]. *PCO* sequence alignment (Figures S2–S6) revealed that most *PCOs* were conserved in *Brassicaceae*, indicating that these duplicated *PCO* genes can still retain the function of their ancestors in these species.

The gene expression pattern of duplicated genes with similar functions may change during the formation of allopolyploids, which takes several typical patterns, including transgressive up/down-regulation, unequal parental contributions, and silencing [40,41]. Although *PCO3* was expanded in *B. napus*, half of them were slightly expressed in different tissues or abiotic stress treatment. According to the results of *PCO3* alignment (Figure S4), *BnaA06G0127100ZS* was derived from *BraA06G014250*, *BnaC05G0155300ZS* was derived from *Bo5G025500*, and the remaining four genes may be generated by mutation or amplification of DNA fragments during the evolution of polyploid. The expression patterns of *BnaA06G0127100ZS* and *BnaC05G0155300ZS* maintained their expression patterns in two diploid progenitors. In addition, the expression of *BnaC04G0074400ZS* and *BnaC05G0283800ZS* responded to abiotic stress (Figure 8, Figure 9 and Figure 10). It indicated that the newly generated genes may contribute to phenotypic differences between allopolyploids and their parental species under abiotic stress conditions.

It has been studied that transcription factors (TFs) bind to cis-regulatory DNA sequences at the 5’ upstream end of genes to activate or repress gene expression [42,43]. In general, genes containing stress response elements in their promoter region are likely to be regulated by stress related TFs [25,31,39,44,45]. It is reported that HSFB2b directly binds to *GmC4H* and *GmCHS3* to regulate the gene expression in response to salt stress, since there are HSEs (Heat Shock Elements) in the promoter regions of the *GmC4H* and *GmCHS3* [46]. It was showed that hormone-responsive elements and environmental stress-related elements were distributed on the *BnaPCO* promoters (Table S2). Combined with the expression data with abiotic stress treatment, *BnaPCO* expression was regulated by various stress responses, especially drought, freezing, cold and waterlogging stress (Figure 8, Figure 9 and Figure 10). However, the expression level was different under different stress conditions. For instance, there were seven anaerobic-induction cis-elements on the promoter region of *BnaC04G0395100ZS*, but the degree of waterlogging induced expression was much different between leaves and roots (Figure 9 and Figure 10). As chromosome structure, histone modification, DNA methylation, transcriptional factors, cis-elements and other regulators work together to regulate gene expression [43], more studies are needed to explore the regulation mechanism of *PCO* expression under abiotic stress.

Oxygen homeostasis is critical for crop development, and hypoxia in plants is typically a consequence of reduced O_2_ diffusion under conditions of waterlogging or submergence [24,47,48,49]. The quality and yield of rapeseed are seriously affected by waterlogging stress in China [28,49]. It is reported that the response to hypoxia in rice, Arabidopsis and barley is mediated by the group VII ETHYLENE RESPONSE FACTORs (ERF-VIIs) [4,8,9,10,19]. Moreover, the *PCOs* directly link O_2_ availability to ERF-VII stability and anaerobic adaptation, leading to the suggestion that they act as plant O_2_ sensors [6,7,11]. There were multiple anaerobic-induction cis-elements distributed on the *BnaPCO* promoters, and *BnaPCO* expression were induced by waterlogging stress after 6 h treatment both in leaves and roots. According to the expression results (Figure 9 and Figure 10), it suggested that *BnaPCO* was a vital component, connecting environmental stimulus with cellular and physiological response and *BnaPCO2* could be a potential target for improving waterlogging stress tolerance. Furthermore, H164 and D176 were in the AtPCO4 active site [12] and it was conserved in BnaPCO4 as shown in Figure S7. It implied that the function of BnaPCO in catalyzing ERF-VIIs could be conserved and targeting *PCOs* will be an effective way to improve the rapeseed tolerance to waterlogging stress by manipulating their O_2_ sensitivity and/or substrate specificity.

## 4. Materials and Methods

### 4.1. Identification of the PCO Gene Family

The protein and nucleotide sequences of *AtPCOs* were obtained with TAIR (https://www.arabidopsis.org/, accessed on 10 June 2022). AtPCO proteins were used as query sequences to search for the PCO proteins of *B. napus*, *B. rapa* and *B. oleracea* using BLASTp (E-value < 1 × 10^−5^) in BARD (http://www.brassicadb.cn/#/BLAST/, accessed on 10 January 2023; protein databases were Brana ZS V2.0 pep, Brara Chiifu V3.5 pep and Braol JZS V2.0 pep, respectively). ExPASy [50] was used to investigate the physical and chemical properties of these PCO proteins.

### 4.2. Phylogenetic Analysis, Chromosomal Locations and Syntenic Relationship

ClustalW was used to align the multiple sequences of all PCO proteins (from *Arabidopsis thaliana*, *B. napus*, *B. rapa* and *B. oleracea*), and MEGA 11 was used to build a phylogenetic tree using the neighbor-joining (NJ) phylogenetic technique with 1000 bootstrap replicates. *Arabidopsis_thaliana*.TAIR10.dna (genome) and *Arabidopsis_thaliana*.TAIR10.gff3 (annotation information) were downloaded from the public database in https://www.arabidopsis.org/ (accessed on 10 January 2023). The genome and annotation information of *B. rapa*_Chiifu_V3.5, *B._oleracea*.BOL, and *B. napus* ZS11 were downloaded from the public database in https://yanglab.hzau.edu.cn/BnIR (accessed on 15 January 2023) [51]. TBtools version 1.116 was used to examine the gene chromosomal localization and syntenic relationship with the genome data, gff3 files and multiple synteny plot tool kit following the software instruction [30].

### 4.3. Analysis of Gene Structure, Motif Composition and Cis-Element

The motifs of BnaPCO proteins were predicted using the MEME v5.5.2 [29]. The number of motifs should not exceed 8. The distribution of motifs occurs zero or one time in each sequence. In order to investigate the structural characteristics of the *BnaPCOs*, the gff3 file (ZS11.annotation.gff3) was downloaded from the *B. napus* database (http://cbi.hzau.edu.cn/cgi-bin/rape/download_ext, accessed on 15 January 2023), which has the annotation information of the *B. napus* genome. The position information of introns and exons is obtained from the gff3 file. Meanwhile, the motif information was submitted to TBtools to graphically display gene structures and motif distributions.

To identify the cis-element of *BnaPCOs*, TBtools was used to obtain the 2000 bp sequences in front of the genomic CDS. Then, the PlantCARE [33] was used to predict the cis-elements on these promoters. Thus, the number and types of different cis-acting elements in *BnaPCOs* were classified and visualized with TBtools.

### 4.4. Plant Materials and Treatments

Zhongshuang 11 (ZS11, a semi-winter cultivar of *Brassica napus* is widely planted in Southern China and the genome sequence is available) seeds were germinated on filter paper, and the seedlings were then transplanted into pots with soil and nurtured in a growth chamber for four weeks (23 °C, 16 h light/8 h dark cycle, a relative humidity of 60%, 300 μmol m^−2 ^s^−1^ light intensity). The waterlogging treatment was performed as previously described [27]. The pots of 4-week-old seedlings were placed in a 28 cm × 14 cm × 14 cm container filled with water and the water level was maintained at approximately 2 cm above the soil surface. Control plants (CK) remained well-watered throughout the experiment.

### 4.5. RNA-Seq and Heat Map Analysis of the PCO Transcriptome Data

Leaf and root samples of CK, 6 h treatment and 12 h-treatment were collected for RNA isolation and total RNA isolated using Trizol Reagent (Invitrogen, Carlsbad, CA, USA) according to the manufacturer’s introductions. There were three biological replicates for each sample. Total amounts and integrity of RNA were assessed using the RNA Nano 6000 Assay Kit of the Bioanalyzer 2100 system (Agilent Technologies, Santa Clara, CA, USA). A total of 1 µg total RNA per sample was used as input material for the lncRNA library preparation. Strand-specific libraries were generated using NEBNext^®^ UltraTM RNA Library Prep Kit for Illumina^®^ (NEB, Ipswich, MA, USA) following the manufacturer’s recommendations and index codes were added to attribute sequences to each sample. RNA Sequencing was performed by the Illumina NovaSeq 6000 (Novogene, Beijing, China). Hisat2 v2.0.5 was used to map the reads to the reference genome and the gene expression level was determined by FPKM (number of Fragments Per Kilobase of transcript sequence per Millions base pairs sequenced) calculation [52]. Differential expression analysis between CK and waterlogging treatment was performed using the edgeR R package. The *p* values were adjusted using the Benjamini and Hochberg method. Significantly differential expression genes were screened based on the following criteria: Corrected *p*-value < 0.05.

The expression patterns of *BnaPCOs* in different tissues and other abiotic stress treatment were obtained from the BnTIR (*Brassica napus* transcriptome information resource) database [37]. The public expression data of *AtPCOs*, *BraPCOs* and *BoPCOs* were obtained from Arabidopsis eFP Browser data, RNA-seq data of *B. rapa* (GSE43245) [35] and RNA-seq data of *B. oleracea* (GSE42891) [36]. All the expression data were standardized based on a log2 scale, and clustered and visualized with TBtools.

### 4.6. Quantitative Real-Time RT-PCR (qRT-PCR) Analysis

To verify the *PCO* gene expression in different tissues, samples of root, stem, leaf, flower, silique and seed from Arabidopsis and *B. napus*, and samples of root, stem and leaf from *B. rapa* and *B. oleracea* were collected. Leaf and root samples of *B. napus* under waterlogging stress treatment with 6 h and 12 h were collected as well. Total RNA was extracted using MolPure^®^ Plant RNA Kit according to manufacturer instructions (Yeasen, Shanghai, China). The first strand cDNA was synthesized by Hifair^®^ III 1st Strand cDNA Synthesis SuperMix for qPCR (gDNA digester plus) (Yeasen, Shanghai, China). Then, the gene relative expression was detected by qRT-PCR assay using PerfectStart^®^ Green qPCR SuperMix (TransGen, Beijing, China) and a CFX96™ Real-Time PCR Detection System (BIO-RAD, Hercules, CA, USA). Gene expression was normalized to *AtActin2*, *BraGAPDH*, *BoActin* and *BnaActin7,* respectively [27,53,54,55]. Relative gene expression values were calculated with the ΔΔCt method. Experiments were performed with three biological replicates. Primers for qRT-PCR were listed in Table S6.

## 5. Conclusions

In this study, we identified 20, 7 and 8 PCO (Plant Cysteine Oxidase) proteins in *B. napus*, *B. rapa* and *B. oleracea*, respectively, by exploring the important role of *PCO* genes in *Brassicaceae* plants. Collinearity analysis shows that the *PCO* gene family was relatively conserved in evolution of *B. rapa*, *B. oleracea*, Arabidopsis, and *B. napus*. However, there were mutations or duplications of *PCO3* and *PCO4* during homologous evolution process. The cis-elements that regulate hormone response and response to abiotic stresses were found in the *BnaPCO* promoters. In addition, we found that the *Bna/Bra/BoPCO* genes were expressed differently in different tissues at different developmental stages. Remarkably, *BnaPCO2s* were significantly induced after waterlogging treatment, which was consistent with the cis-element analysis and previous studies. *BnaPCO2* could be the potential target for waterlogging tolerance improvement. This study provides a foundation for further understanding the biology and stress response functions of PCO family genes in *B. napus*.

## Figures and Tables

**Table 1:** List of *PCO* genes identified in *B. rape*, *B. oleracea* and *B. napus*.

	Gene ID	Nucleotide Length (bp)	Amino Acid	Molecular Weight (KD)	PI	Genome Location	Number of Introns	Number of Exons
*PCO1*	*BnaA03G0061400ZS*	897	299	33.22	7.5	ChrA03: 2,917,715–2,919,518	4	5
	*BnaA10G0211400ZS*	924	308	34.18	8.01	ChrA10: 22,378,464–22,380,486	4	5
	*BnaC03G0070600ZS*	879	293	32.75	7.77	ChrC03: 3,619,461–3,621,089	4	5
	*BnaC09G0512300ZS*	771	257	28.66	5.91	ChrC09: 61,478,635–61,483,755	4	5
	*Bo3G010080*	876	292	32.62	7.52	3,923,348–3,924,992	4	5
	*Bo9G165400*	918	306	33.96	8.78	60,734,007–60,728,419	4	5
	*BraA03G006660*	897	299	33.3	7.5	2,874,949–2,876,803	4	5
	*BraA10G024330*	717	239	26.63	5.14	16,636,583–16,634,714	4	5
*PCO2*	*BnaA04G0111100ZS*	846	282	31.35	8.21	ChrA04: 12,952,829–12,954,006	4	5
	*BnaC04G0395100ZS*	858	286	31.75	8.21	ChrC04: 52,079,647–52,080,830	4	5
	*Bo4G139190*	858	286	31.75	8.21	46,652,407–46,653,590	4	5
	*BraA04G013090*	846	282	31.34	8.02	9,741,294–9,742,752	4	5
*PCO3*	*BnaA06G0127100ZS*	831	277	30.61	5	ChrA06: 7,448,257–7,449,546	4	5
	*BnaC04G0074400ZS*	246	82	8.65	4.39	ChrC04: 6,512,883–6,514,191	2	3
	*BnaC05G0155300ZS*	831	277	30.62	5.01	ChrC05: 9,956,060–9,957,351	4	5
	*BnaC05G0283800ZS*	318	106	11.58	4.17	ChrC05: 24,420,297–24,421,163	1	2
	*BnaC05G0471900ZS*	387	129	14.3	8.6	ChrC05: 52,043,598–52,044,332	1	2
	*BnaC09G0521600ZS*	387	129	14.23	8.37	ChrC09: 62,173,896–62,174,630	1	2
	*Bo5G025500*	834	278	30.67	5.01	9,490,230–9,491,521	4	5
	*BraA06G014250*	831	277	30.55	4.89	7,467,637–7,469,108	4	5
*PCO4*	*BnaA03G0207500ZS*	624	208	23.51	6.5	ChrA03: 10,842,878–10,844,151	3	4
	*BnaA05G0034400ZS*	729	243	27.11	6.03	ChrA05: 1,925,460–1,927,173	4	5
	*BnaC03G0244400ZS*	636	212	23.54	8.04	ChrC03: 14,957,859–14,959,210	4	5
	*BnaC04G0037200ZS*	717	239	26.81	6.42	ChrC04: 3,448,037–3,452,967	4	5
	*Bo3G036180*	636	212	23.53	8.04	15,839,677–15,841,028	4	5
	*Bo4G017390*	717	239	26.75	6.23	3,496,211–3,497,884	4	5
	*BraA05G003220*	717	239	26.72	6.42	1,719,860–1,722,377	4	5
*PCO5*	*BnaA04G0023400ZS*	729	243	27.23	6.84	ChrA04: 1,525,382–1,527,222	4	5
	*BnaA09G0537000ZS*	729	243	27.13	6.59	ChrA09: 56,193,470–56,194,841	4	5
	*BnaC04G0289700ZS*	729	243	27.22	6.78	ChrC04: 39,729,996–39,732,404	4	5
	*BnaC08G0384200ZS*	729	243	27.2	6.5	ChrC08: 44,790,558–44,792,029	4	5
	*Bo4G108280*	729	243	27.22	6.78	34,730,028–34,731,729	4	5
	*Bo8G092800*	729	243	27.2	6.5	41,444,747–41,446,184	4	5
	*BraA04G002890*	729	243	27.22	6.99	1,612,409–1,614,775	4	5
	*BraA09G050770*	729	243	27.12	6.59	36,671,235–36,668,985	4	5

## Data Availability

The clean reads of RNA-seq in this paper have been deposited in the National Genomics Data Center [56,57] (GSA: CRA010912). All relevant data are available from the corresponding author on request (mani@caas.cn).

## Supplementary Materials

The following supporting information can be downloaded at: https://www.mdpi.com/article/10.3390/ijms241411242/s1.
