# Supplementary material for: Genome-Wide Identification and Analysis of the Plant Cysteine Oxidase (PCO) Gene Family in Brassica napus and Its Role in Abiotic Stress Response
Source: Int J Mol Sci. 2023 Jul 8;24(14):11242. doi: 10.3390/ijms241411242 (PMC10379087; doi:10.3390/ijms241411242)
Supplement: Supplementary file 1 [file ijms-24-11242-s001.zip › Supplemental Figure.pptx]

## Slide 1
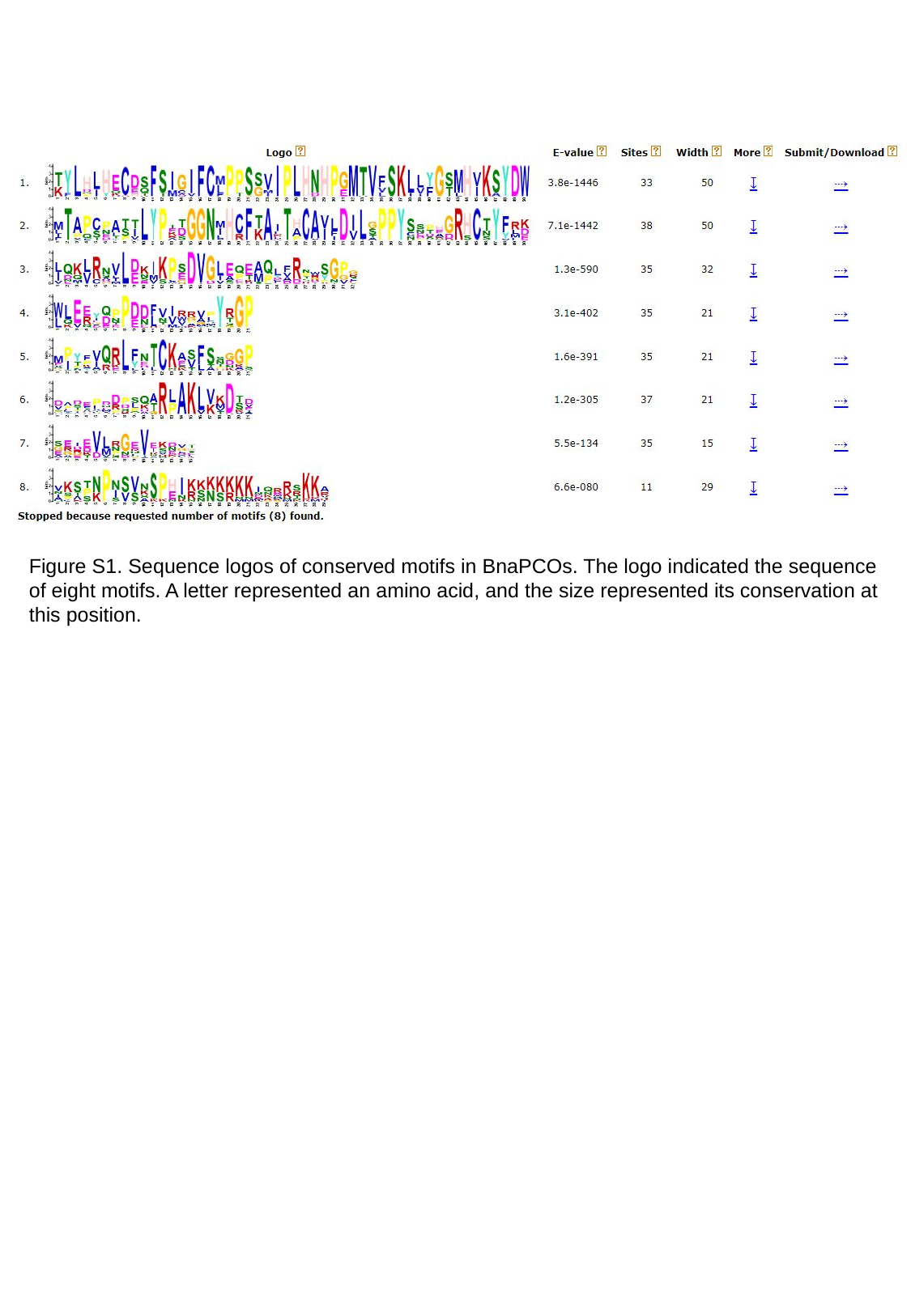

Figure S1. Sequence logos of conserved motifs in BnaPCOs. The logo indicated the sequence of eight motifs. A letter represented an amino acid, and the size represented its conservation at this position.

## Slide 2
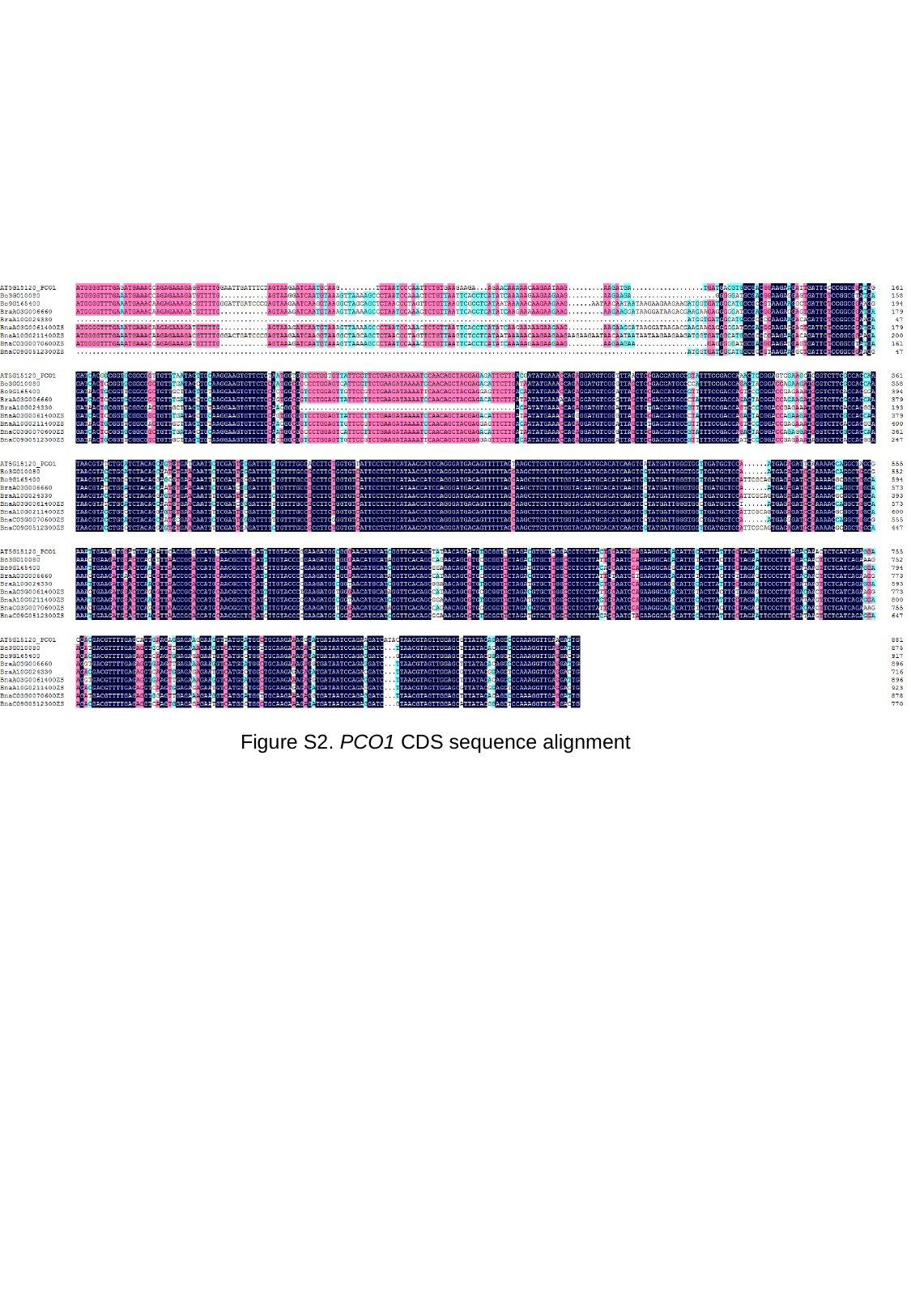

Figure S2. PCO1 CDS sequence alignment

## Slide 3
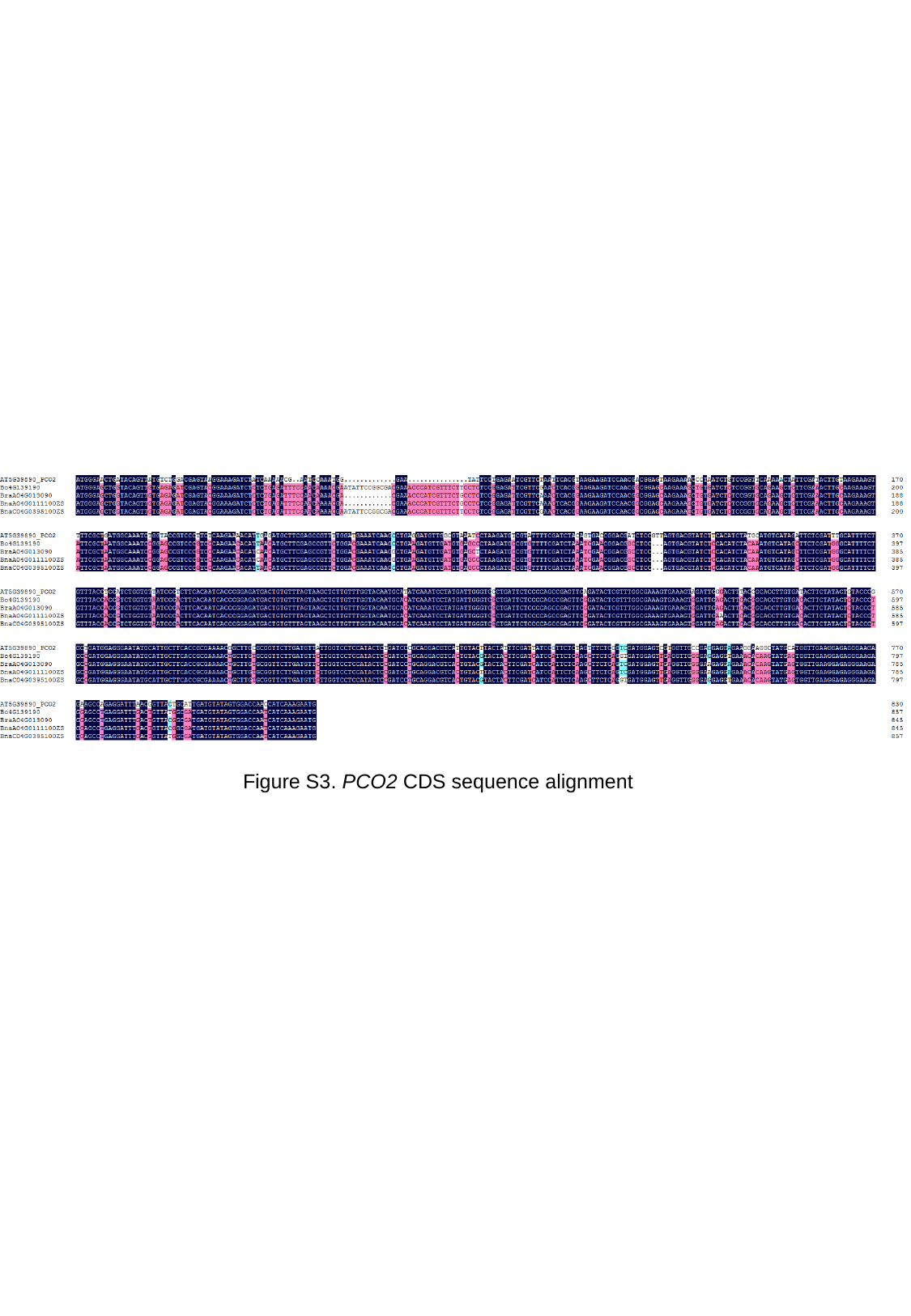

Figure S3. PCO2 CDS sequence alignment

## Slide 4
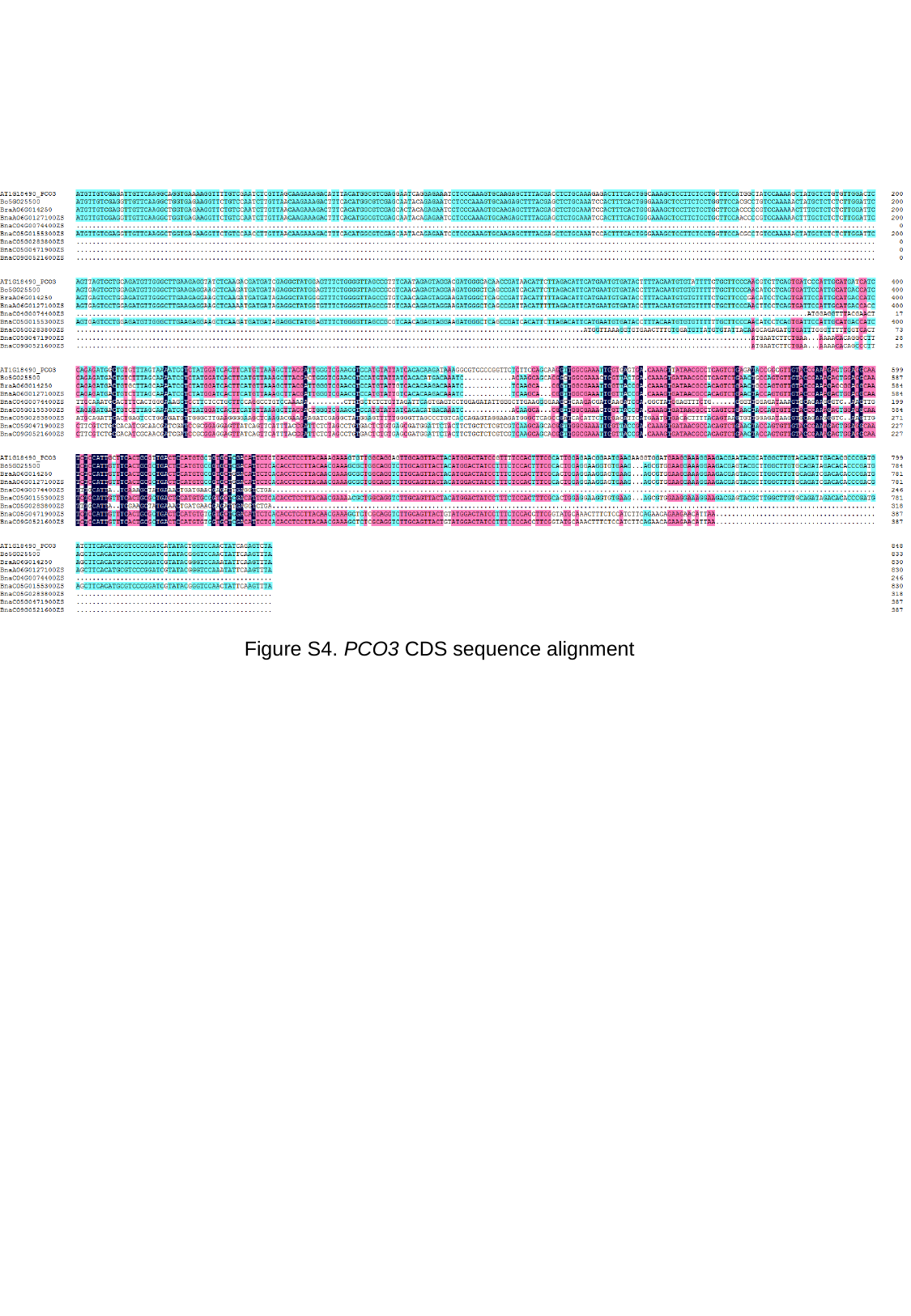

Figure S4. PCO3 CDS sequence alignment

## Slide 5
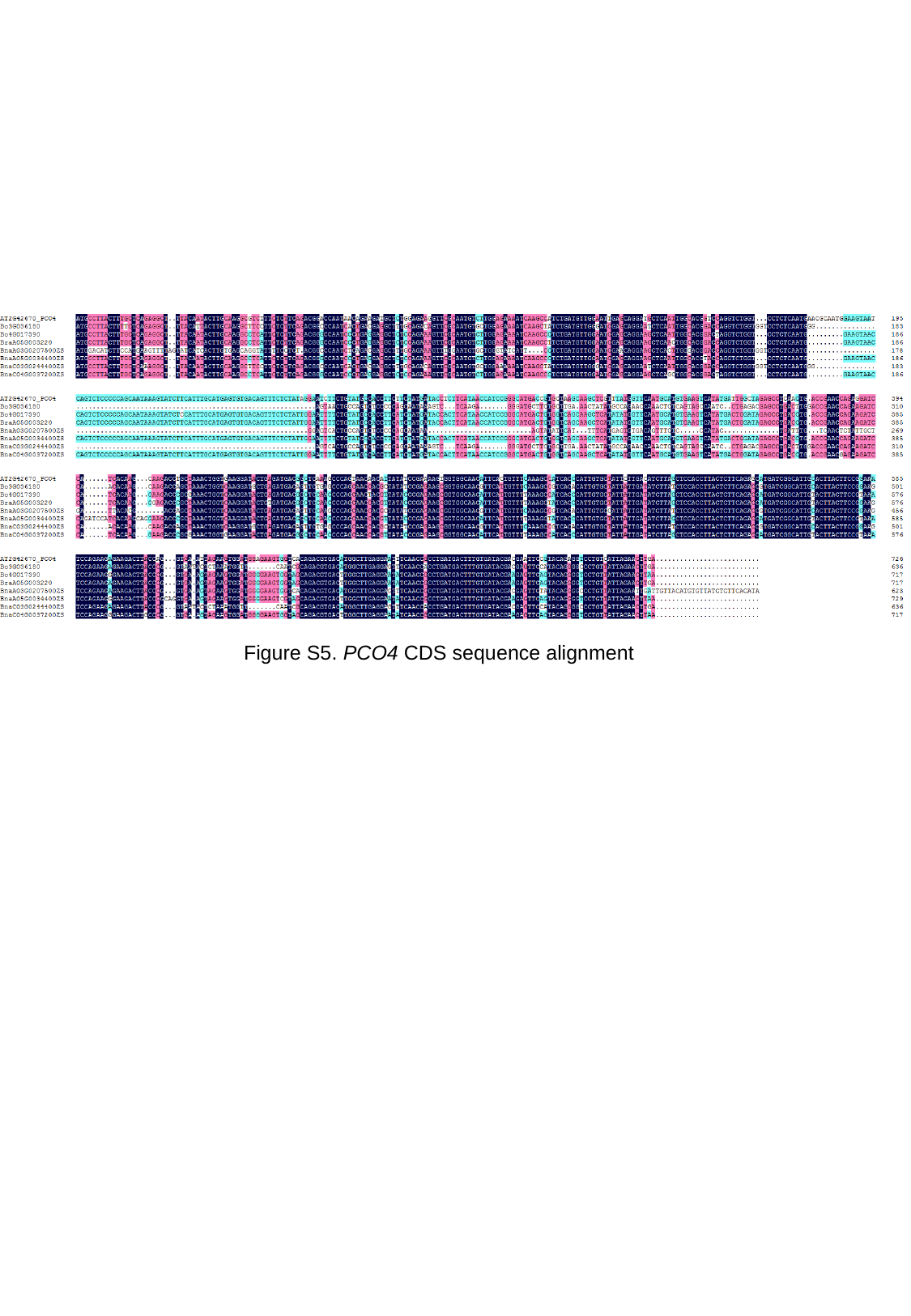

Figure S5. PCO4 CDS sequence alignment

## Slide 6
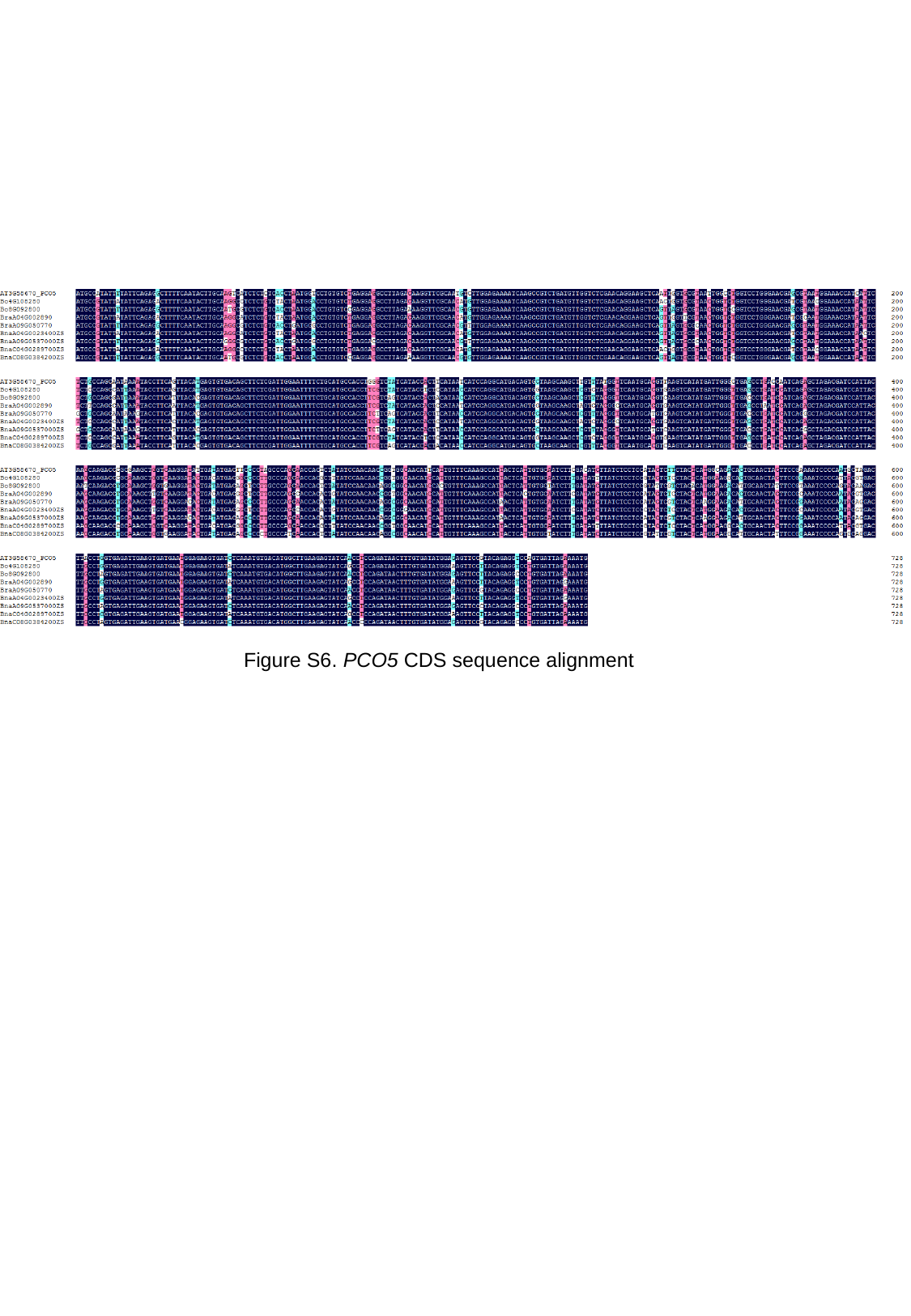

Figure S6. PCO5 CDS sequence alignment

## Slide 7
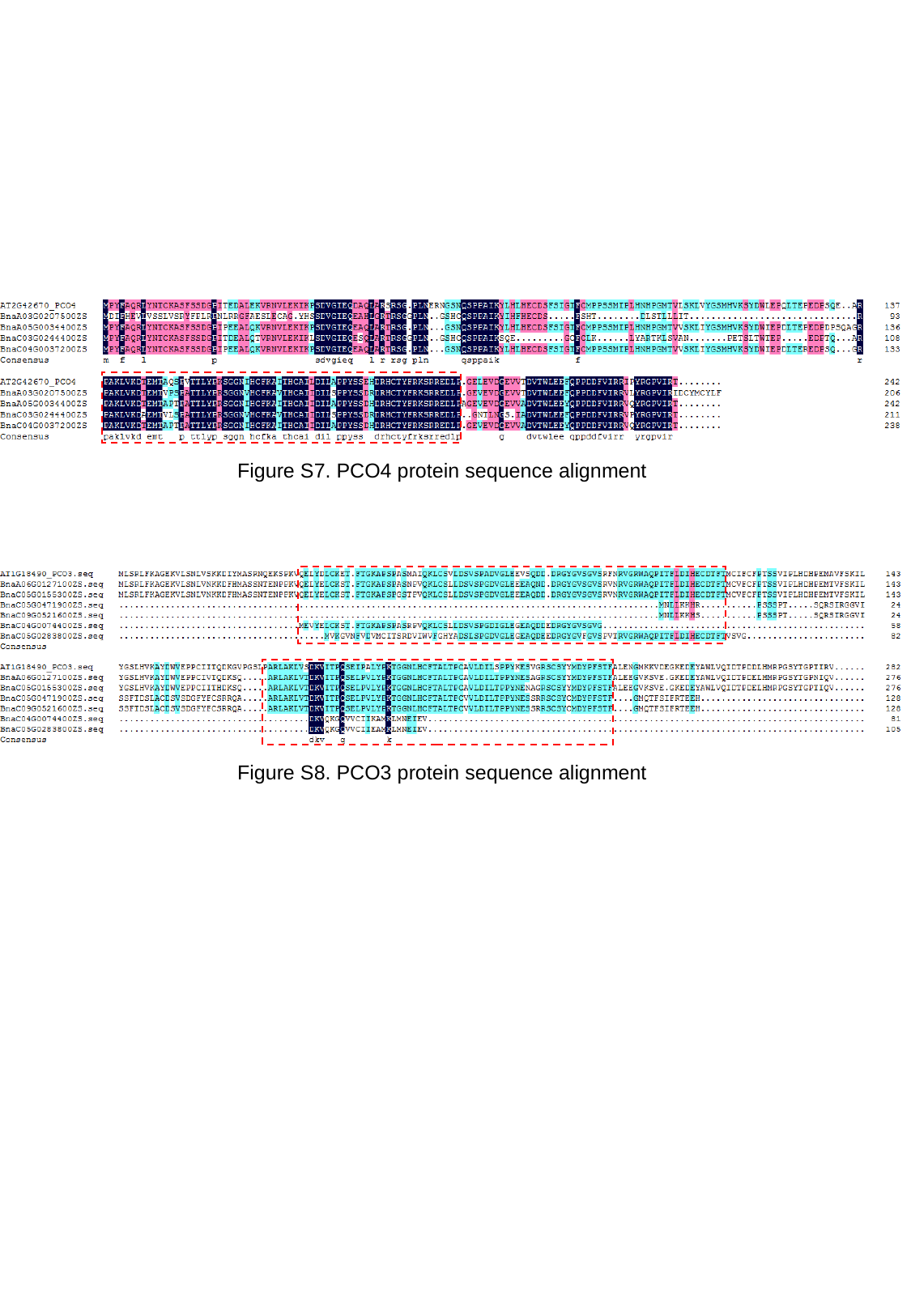

Figure S7. PCO4 protein sequence alignment
Figure S8. PCO3 protein sequence alignment

## Slide 8
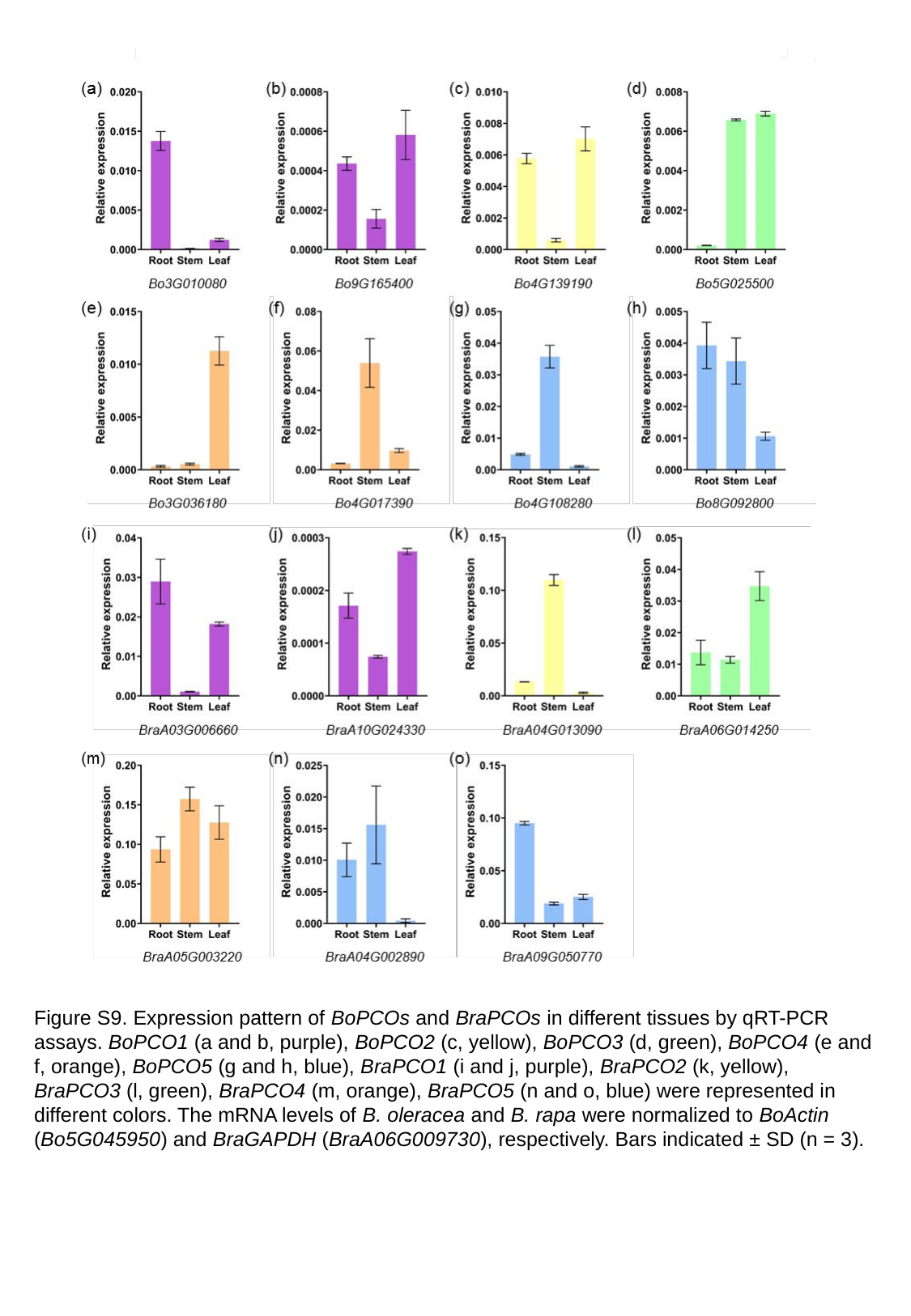

Figure S9. Expression pattern of BoPCOs and BraPCOs in different tissues by qRT-PCR assays. BoPCO1 (a and b, purple), BoPCO2 (c, yellow), BoPCO3 (d, green), BoPCO4 (e and f, orange), BoPCO5 (g and h, blue), BraPCO1 (i and j, purple), BraPCO2 (k, yellow), BraPCO3 (l, green), BraPCO4 (m, orange), BraPCO5 (n and o, blue) were represented in different colors. The mRNA levels of B. oleracea and B. rapa were normalized to BoActin (Bo5G045950) and BraGAPDH (BraA06G009730), respectively. Bars indicated ± SD (n = 3).
